# Impact of immigrants on a multi-agent economical system

**DOI:** 10.1371/journal.pone.0197509

**Published:** 2018-05-24

**Authors:** Léa Kaufmann, Ranaivo Razakanirina, Derek Groen, Bastien Chopard

**Affiliations:** 1 Computer science department, University of Geneva, Geneva, Switzerland; 2 Department of computer science, Brunel University London, London, United Kingdom; Universidad Veracruzana, MEXICO

## Abstract

We consider a multi-agent model of a simple economical system and study the impacts of a wave of immigrants on the stability of the system. Our model couples a labor market with a goods market. We first create a stable economy with *N* agents and study the impact of adding *n* new workers in the system. The time to reach a new equilibrium market is found to obey a power law in *n*. The new wages and market prices are observed to decrease as 1/*n*, whereas the wealth of agents remains unchanged.

## Introduction

Understanding the impact of massive immigration is an important challenge today. Its impact is often difficult to quantify, and can be perceived very differently from a human, societal or economical point of view.

The number of international migrants worldwide has continued to grow rapidly over the past fifteen years, reaching 244 millions in 2015, up from 222 millions in 2010 and 173 million in 2000 [1]. Among them, there are many refugees who are forced to move away from their original location. According to the United Nations High Commissioner for Refugees (UNHCR), by the end of the year 2015, 65.3 million individuals were forcibly displaced worldwide as a result of persecution, conflict, generalized violence, or human rights violations [2]. Global warming may also become a primary factor of forced immigration, leading to disruptions of monsoon systems, droughts of unprecedented severity and coastal flooding [3]. Migration routes and irregular immigration are causing additional social and economical issues. There are an important component of the global problem. For a link to this wider context, see for instance [4–7].

Here, we chose to focus our discussion on issues that arise around the countries of destination. A central question is how this new immigrant population integrates within the economic environment of the hosting country on the long term. This is obviously not a simple question as it depends on many factors that are hard to know and control. We can however explore such a question in a simplified theoretical context, within the framework of complex systems, focusing for example on the effects of immigration on important elements such as wages, prices and wealth.

Socio-economical systems are now commonly described at the microlevel, as dynamical and/or multi-agent systems (see for instance [8–14]). Each agent represents an idealized person (trader, worker, …) or fundamental entity (firm, market maker, …), obeying given behavioral rules, usually with stochastic components. Out of the interactions between many of these agents, collective properties emerge, that define the state and evolution of the corresponding society and/or its economy.

Here we present a numerical model which mimics a system of persons working for firms, producing goods that are then sold to the market. The workers, thanks to the salary they receive from the firms they work for, can buy these goods. This produces a profit to the firm, which is then used to pay the salary.

Here we consider the situation of immigrants when they are ready to work in the hosting economy. That is, we disregard the time needed fot getting a work permit or to go through the administrative immigration procedures (see for instance [15–17] for a discussion of these points). The social cost of these steps could of course be added to a more elaborated model, but this is for now beyond the scope of the present work. Our focus is primarily on how an economical system will absorb immigrants, neglecting their social integration (cost of crime resulting from and unsucessful intgeration) and that of their children (cost related to their education). Interesting references on this topic can be found, for instance in [18, 19]. We hope that the model we propose here will once be extended to include these indirect social aspects. But, our goal now is the keep the model as simple as possible, without adding to many hard to specify parameters or processes. For this reason our study only includes a simple labor-good market.

More precisely, the main question we want to investigate here is to quantify the impact of perturbing a given economical system by the arrival of new workers. The main effect of such a perturbation is to create a new equilibrium situation, with new salary, wealth and price levels. But this new equilibrium is only reached after a period of time. The main control parameters we will consider are the size of the initial economy and the size of the perturbation, given by the number of newly introduced workers.

Our results show that the time to stabilize the economy after a perturbation of size *n* increases as a power law of *n*. We also obtain that the new equilibrium price is lower, as well as the hourly salary. The decrease in hourly salary is in line with existing literature, as reported for example by Borjas et al. [20, 21]. The amplitude of these changes is more pronounced as *n* increases. The drop of wage and price is also found to follow a power law. However, the wealth of the workers and firms, as well as purchase power remain constant, up to fluctuations across the agents.

## Model

The model we consider here has been proposed and developed in [22–26]. It is based on the interactions between agents sitting at the nodes of a given complex network (whether random, scale-free or from observational data). Agents are of two types, called here “workers” or “firms”. The role of the workers is to be buyer-employee and that of the firms is to be seller-employer. In what follow we will use the index *i* to denote a worker/buyer and the index *j* to denote a firm/seller.

**Fig 1:**
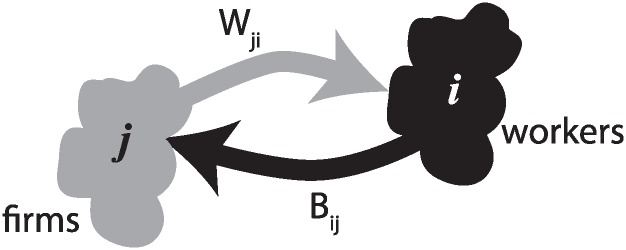
Graphical sketch of the bipartite graph in which a worker is connected to a firm through *W*_*ij*_ for employee-employer relation, and through *B*_*ij*_ for the buyer-seller relation.

Each agent interacts with its neighbors, as specified by two given adjacency matrices *B*_*ij*_ and *W*_*ij*_. There are two of them as there are two relations, “buyer-seller” or “employee-employer”. We define as *W*(*i*) the set of all firms that a worker *i* works for and *W*^−1^(*j*) the set of all workers employed by firm *j*. Likewise, we denote by *B*(*i*) the set of firms agent *i* buys from, and by *B*^−1^(*j*) the set of workers/consumers firm *j* is selling goods to. Fig 1 depicts the bipartite graph that describes the worker-firm relation.

From the definitions of *B*(*i*) and *B*^−1^(*j*) we necessarily have that, for any quantity *x*_*ij*_,

$$\begin{array}{c} \sum_{j}\sum_{i\in B^{-1}\left(j\right)}x_{ij}=\sum_{i}\sum_{j}B_{ij}x_{ij}=\sum_{i}\sum_{j\in B(i)}x_{ij}, \end{array}$$ (1)

where the sum over *j* means the sum over all firms and the sum over *i* corresponds to the sum over all workers. The same rule obviously holds when replacing *B* by *W*.

Note that before starting a simulation, these networks are tuned so that they obey some minimal constraints: a worker/consumer must at least have one employer and buy from at least one firm. A firm must have at least one employee and one client. We do not explicitly impose that the graphs defined by the matrices *W*_*ij*_ and *B*_*ij*_ are connected, but they are built here randomly with a probability of a link which is large enough to produce connected graphs.

The dynamics implement the coupling of two markets, namely a labor market and a goods market. A link between a firm and a worker (or buyer) is cut whenever the interaction turns out to be unprofitable for the latter. In the presented implementation, no new links are created.

Note that in [26], a *credit market* model has also been introduced to study the stability of a market in which banks lend money for a given time to other agents of the market. There, the behavior of the total debt is considered as well as the possibility to control the market away from bankruptcy.

### The labor market

In the *labor market* a worker *i*, at each iteration *t*, spends *h*_*ij*_(*t*) working hours at firm *j*, for all *j* ∈ *W*(*i*). The sum of all the time spent in these firms amounts to the number of working hours *h* per time period. A time period corresponds to an iteration of the model and can be arbitrarily chosen. It could be a week, or a month. Here, we simply choose it as a day, so that we fixed *h* to 8 daily hours of work,

$$\begin{array}{c} h=\sum_{j\in W(i)}h_{ij}=8\;\forall \;\text{worker}\;i. \end{array}$$

As discussed below, the distribution *h*_*ij*_ of time depends on the hourly salary offered by each company *j*.

The total amount

$$\begin{array}{c} H_{j}\left(t\right)=\sum_{i\in W^{-1}\left(j\right)}h_{ij}\left(t\right) \end{array}$$ (2)

of hours received at each iteration by a firm *j* is used to produce an amount of goods *G*_*j*_(*t*) which is here proportional to *H*_*j*_. We choose arbitrarily that each hour of work produces 2 units of goods, that is *G*_*j*_(*t*) = 2 × *H*_*j*_(*t*).

Once produced, these goods will be sold in the *goods market*, as explained below, and will increase the asset *C*_*j*_(*t*) of the firm. A fraction *μ* of this money is then used to pay salaries to the workers. Precisely, an amount *μC*_*j*_(*t*) is distributed among the workers, in proportion to the time they worked for firm *j* at iteration *t*. The salary *s*_*ij*_(*t*) received by worker *i* from firm *j* is denoted as *s*_*i*_(*t*) and is then obtained as

$$\begin{array}{c} s_{ij}\left(t\right)=h_{ij}\left(t\right)\frac{\mu C_{j}}{H_{j}\left(t\right)}=h_{ij}\left(t\right)S_{j}\left(t\right) \end{array}$$ (3)

where *S*_*j*_(*t*) = *μC*_*j*_/*H*_*j*_(*t*) is the hourly salary effectively given by firm *j* at iteration *t*. The total salary *s*_*i*_(*t*) a worker *i* receives at iteration *t* for all the firms he is working for is then

$$\begin{array}{c} s_{i}\left(t\right)=\sum_{j\in W(i)}s_{ij}\left(t\right). \end{array}$$ (4)

As required, the total salary spent by firm *j* for all its employees is *μC*_*j*_(*t*)

$$\begin{array}{c} \sum_{i\in W^{-1}\left(j\right)}s_{ij}\left(t\right)=\frac{\mu C_{j}\left(t\right)}{H_{j}\left(t\right)}\sum_{i\in W^{-1}\left(j\right)}h_{ij}\left(t\right)=\mu C_{j}\left(t\right). \end{array}$$ (5)

Moreover, using (1), we also verify that, indeed,

$$\begin{array}{c} \sum_{i}s_{i}\left(t\right)=\sum_{i}\sum_{j\in W(i)}s_{ij}\left(t\right)=\sum_{j}\sum_{i\in W^{-1}\left(j\right)}s_{ij}=\mu \sum_{j}C_{j}\left(t\right). \end{array}$$ (6)

which confirms that the sum of the salaries is equal to the total spending of the firms.

Depending on the salary received from each firm, a worker will re-adjust the time *h*_*ij*_ he spends at each of them for the next iteration. However, to avoid potential salary oscillations resulting from a brutal redistribution, the decision is based on a time average ${\bar{S}}_{j}$ of the hourly salary over two iterations

$$\begin{array}{c} {\bar{S}}_{j}\left(t\right)=\gamma S_{j}\left(t\right)+\left(1-\gamma \right){\bar{S}}_{j}\left(t-1\right) \end{array}$$

in which we chose *γ* = 0.5. Initially, it is assumed that ${\bar{S}}_{j}\left(0\right)=1$ for all *j*. Note that in the steady state (or equilibrium), we have $S_{j}={\bar{S}}_{j}$.

This memory effect can of course be extended to more time steps, if needed. It is expected to dampen the “ping-pong” effect between agents, but not to remove possible chaotic regimes, as observed in [26].

The new distribution of working hours is then computed as

$$\begin{array}{c} h_{ij}\left(t+1\right)=h\times \frac{{\bar{S}}_{j}\left(t\right)}{\sum_{k\in W(i)}{\bar{S}}_{k}}. \end{array}$$

If there is a large difference among the hourly salary offered by the firms *j* ∈ *W*(*i*), worker *i* decides to break the link with the firm that pays less. The rule we implemented is that if there is a factor larger than 2 between the higher and lower salary that worker *i* can receive, worker *i* will cut the link with the less profitable firm. As a result the adjacency matrix *W*_*ij*_ is updated, as well as *W*(*i*) and *W*^−1^(*j*).

### The goods market

The second part of the model is a *goods market* in which a worker (or consumer) *i* spends an amount *a*_*ij*_(*t*) of its money to acquire goods from all the firms *j* within its buying relations, *j* ∈ *B*(*i*), as specified by the adjacency matrix *B*_*ij*_. Here we assume that this amount *a*_*ij*_ spent to buy goods is a fraction *λ* of the agent’s wealth, *c*_*i*_(*t*), plus the total wage *s*_*i*_(*t*) received for the work done at iteration *t*, as given by Eq (4).

The amount *a*_*ij*_ is inversely proportional to the price *p*_*j*_(*t*) proposed by firm *j*, namely

$$\begin{array}{c} a_{ij}\left(t\right)=\lambda \times \left[c_{i}\left(t\right)+s_{i}\left(t\right)\right]\frac{p_{j}^{-1}}{\sum_{k\in B(i)}p_{k}^{-1}}. \end{array}$$ (7)

Initially, the prices *p*_*j*_(0) are set to 1, so that the worker does not assume any difference between the firms.

The total amount of the purchase of agent *i* is then

$$\begin{array}{c} a_{i}\left(t\right)=\sum_{j\in B(i)}a_{ij}\left(t\right)=\lambda \times \left[c_{i}\left(t\right)+s_{i}\left(t\right)\right] \end{array}$$ (8)

and its wealth is updated as

$$\begin{array}{c} c_{i}\left(t+1\right)=c_{i}\left(t\right)+s_{i}\left(t\right)-a_{i}\left(t\right)=\left(1-\lambda \right)\times \left[c_{i}\left(t\right)+s_{i}\left(t\right)\right]. \end{array}$$ (9)

With this amount *a*_*ij*_(*t*) the worker receives from firm *j* an amount *g*_*ji*_(*t*) of goods. The value of *g*_*ji*_ is computed by the firm *j*, proportional to the money received from all the buyers *k* ∈ *B*^−1^(*j*) and the amount *G*_*j*_(*t*) of goods available

$$\begin{array}{c} g_{ji}\left(t\right)=G_{j}\left(t\right)\frac{a_{ij}\left(t\right)}{\sum_{k\in B^{-1}\left(j\right)}a_{kj}\left(t\right)}. \end{array}$$ (10)

This interaction defines the real price of goods by the ratio of the amount paid to the amount of goods received

$$\begin{array}{c} p_{ji}=\frac{a_{ij}\left(t\right)}{g_{ij}\left(t\right)}=\frac{a_{ij}}{a_{ij}G_{j}}\sum_{k\in B^{-1}\left(j\right)}a_{kj}=\frac{1}{G_{j}}\sum_{k\in B^{-1}\left(j\right)}a_{kj}. \end{array}$$ (11)

The last equality shows that actually *p*_*ji*_ is the same for all buyers *i* and depends only on the firm *j*.

This new price will affect how the buyers will distribute their purchase among the firms at iteration *t* + 1. However, to avoid oscillation effects due to a brutal change of distribution *a*_*ij*_, the decision is based on the price *p*_*j*_(*t* + 1) defined as an interpolation of the real price and the previous price

$$\begin{array}{c} p\left(t+1\right)=\rho \frac{a_{ij}\left(t\right)}{g_{ij}\left(t\right)}+\left(1-\rho \right)p_{j}\left(t\right) \end{array}$$ (12)

where we chose *ρ* = 0.5. Note that in the steady state, the indicative price *p*_*j*_ and the actual one *a*_*ij*_/*g*_*ij*_ are equal.

We also consider for the goods market a rule to cut links that turns non-profitable. If the price ratio between the cheapest and most expensive firm is smaller than 0.5, the worker will stop buying from the expensive one, thus modifying the adjacency matrix *B*_*ij*_.

From the above interaction rules, the wealth of a firm is updated at each iteration, based on the balance of the payment of the salaries and the profit earned from the sales,

$$\begin{array}{c} C_{j}\left(t+1\right)=C_{j}\left(t\right)-\sum_{i\in W^{-1}\left(j\right)}s_{ij}\left(t\right)+\sum_{i\in B^{-1}\left(j\right)}a_{ij}\left(t\right)=\left(1-\mu \right)C_{j}\left(t\right)+\sum_{i\in B^{-1}\left(j\right)}a_{ij}\left(t\right) \end{array}$$ (13)

where we have used (5).

As can be expected this *labor & goods market* model conserves exactly the sum of the wealth of all agents, namely

$$\begin{array}{c} \sum_{i}c_{i}\left(t+1\right)+\sum_{j}C_{j}\left(t+1\right)=\sum_{i}c_{i}\left(t\right)+\sum_{j}C_{j}\left(t\right). \end{array}$$ (14)

To verify these properties, we sum (9) over *i*, using (6)

$$\begin{array}{ccc} \sum_{i}c_{i}\left(t+1\right) & = & \left(1-\lambda \right)\sum_{i}c_{i}\left(t\right)+\left(1-\lambda \right)\sum_{i}s_{i}\left(t\right)\\ & = & \left(1-\lambda \right)\sum_{i}c_{i}\left(t\right)+\left(1-\lambda \right)\mu \sum_{j}C_{j}\left(t\right) \end{array}$$ (15)

where, as before, a sum over *i* concerns the workers and a sum over *j* refers to firms. Similarly, from (13) with (1) and (8)

$$\begin{array}{ccc} \sum_{j}C_{j}\left(t+1\right) & = & \sum_{j}\left(1-\mu \right)C_{j}\left(t\right)+\sum_{j}\sum_{i\in B^{-1}\left(j\right)}a_{ij}\\ & = & \sum_{j}\left(1-\mu \right)C_{j}\left(t\right)+\sum_{i}\sum_{j\in B(i)}a_{ij}\\ & = & \sum_{j}\left(1-\mu \right)C_{j}\left(t\right)+\lambda \sum_{i}\left[c_{i}\left(i\right)+s_{i}\left(t\right)\right]\\ & = & \sum_{j}\left(1-\mu \right)C_{j}\left(t\right)+\lambda \sum_{i}c_{i}\left(t\right)+\lambda \mu \sum_{j}C_{j}\left(t\right) \end{array}$$ (16)

which achieves to verify the conservation of the total wealth in the system.

It is good to note that the above *labor & goods* model is deterministic, except for the initial conditions. Also it has very few tuning parameters: they are the threshold values to cut a link and the fraction *λ* of the wealth each worker invests in buying goods and the fraction *μ* of its wealth that each firm spends in salary.

A simulation is typically started with a given number *N* of worker-buyers and a number *M* << *N* of firms. At time *t* = 0, an initial asset is generated randomly for each agent. As time goes on, the above interactions rules create a circulation of money and goods, which evolves to a steady state where the salary and price no longer change, unless an external perturbation appears.

We refer the reader to [22–25, 27] for more details on the technical implementation of this *labor & goods* model, as well as for small variants of the model.

## Results

In this section we perform numerical simulations with our *labor & goods* model. In order to explore the effect of a wave of immigration in a country with a stable economy, we investigate the impact of an external perturbation on the market after it has reached its equilibrium state.

We will consider a stable economy of size *N*, and then add *n* new workers. Our main question is how long does it take to absorb these extra *n* agents and reach an equilibrium situation, as a function of *N* and *n*. Of course, we do not expect precise time scales, but rather an answer showing whether the effect of the perturbation is linear, quadratic, exponential, etc in its size. In addition to the time to re-stabilize the system, we will also analyze the change in macro-economical variables, such as price, salary and asset.

We shall first present the results of the numerical simulation of the model. Then we will also develop a *mean-field* theoretical approach, that is an approach where all the dynamics is reduced to two representative agents.

### Numerical simulation: The reference economy

The numerical simulation we consider here contains *N* = 120 workers and *M* = 30 firms. The size of this economy turns out to be large enough to avoid small size effects. Indeed, as visible in Table 1, larger systems (e.g. *N* = 800 and *M* = 200) behave very similarly provided that the numbers of firms each worker buys from or works for remain the same, *i.e.* by adjusting the probabilities to creates the workers/firms and buyers/sellers links. This can be understood intuitively if we think that the larger system is the concatenation of smaller ones, with the same topological properties. Whereas the average properties are the same in the small and large systems, the range of variation of prices, salaries and wealth among the agents increases if the size of the system gets larger.

**Table 1:** Key characteristics of our reference economies, prior to adding immigrants.

	*N* = 120, *M* = 30	*N* = 800, *M* = 200
**convergence time**	20	20
**hourly salary**	17.7±1	17.7±1.3
highest salary	20.2	21.5
lowest salary	16.1	14.9
**price**	8.8±0.5	8.9±0.7
highest price	10.1	10.7
lowest price	8.0	7.5
**wealth of workers**	68.8±0.9	68.8±1.3
**wealth of firms**	1125±99	1124.6±114
richest firm	1299.8	1432.3
poorest firm	831.2	783.5

Our goal is not to systematically explore all possible values of the ratio *N*/*M*, but rather to produce an initial stable economical system that we can further perturb by adding *n* immigrants. We may of course expect that the ratio of firms to workers will affect the way the *n* immigrants are integrated in the system. We leave this question for a forthcoming study, focusing mostly on a qualitative identification of the typical modifications resulting from a sudden change of *N* into *N* + *n*.

The *N* workers and *M* firms are here connected with a random network: each pair of worker-firm is linked with probability 0.6 for the purchase relation (*goods market*) and with a probability 0.3 for the work-salary relation. This means that on average, a worker initially shares his time among 9 companies and buys from 18.

At this stage we only consider a random graph, as this paper focuses on conceptual ideas. Also note that building a synthetic bipartite scale free network is less well defined than for graphs with only one type of nodes.

Another remark concerns the number of companies a agent works for. This number may appear large in the present study, but it can easily be adjusted by the probability of creating a link. However, we believe that having several employers is not unrealistic. For instance it can represent people who are independant and have several contract with different firms. Another interpretation is that the firms of the model are actually part of a larger company for which the workers have to complete different tasks.

As already mentioned, we have chosen the following parameters in the present study: each worker works 8 hours per iterations, and each working hour produces 2 units of goods to the company. The investment parameters are taken as *λ* = *μ* = 1/2.

The initial wealth of each worker *i* is *c*_*i*_(0) = 100, whereas the initial wealth of each firm *j* is *C*_*j*_(0) = 1000. We observe that repeating the *labor & goods* simulation several times, with a new instance of the interacting network lead to the same qualitative results, namely a convergence to a steady state after about 20 iterations.

We have then selected one specific network configuration and run the model to convergence. Its properties are summarized in Table 1. This will be our reference economy, one among many possibilities, that will then be perturbed by the arrival of immigrants. The number of iterations (20 in this case) to reach the steady state is to be understood as the reference time required for an economy to stabilized from a random initial condition. Below, we compare it to the time the perturbed systems takes to find its new equilibrium sate.

Although not shown in Table 1, we observe in this reference system that the companies that offer the highest price also offer the best salary, and the firms with the lowest price also offer the lowest salary. In addition, the wealth of the companies is higher for those offering a lower salary or a higher price, and lower for those offering a lower price or a higher salary.

Globally, all workers are poorer than they were initially, but only 3 out of the 30 firms have lost money upon reaching the equilibrium state. The wealth of the firms is strongly correlated with the sum of the number of workers and consumers connected to them. However, the price is not correlated to the number of consumers, and the hourly salary only has a weak negative correlation with the number of workers in a company [27].

### Numerical simulation: The impact of immigrants

In this section we study the impact on our reference economy of adding *n* immigrants, for various values of *n*. We mostly discuss our results for our reference system with *N* = 120 workers and *M* = 30 firms. The system with *N* = 800 workers and *M* = 200 firms gives the same results, for the same ratio *n*/*N* of added workers.

We added *n* new workers to the reference system all at once. The new workers received the same initial wealth as the native worker, namely *c*_*i*_ = 100 units of money in the present case. The number *M* of firms stays constant, as we here consider the case where the immigrants are employees and do not create new companies. The *n* new workers are connected randomly to the *M* firms, with the same probabilities as used in the original system, namely a probability 0.6 to be connected to a firm for the buy/sell relation and a probability 0.3 to be connected for the work/salary relation. We considered 50 different realizations of connecting the *n* new agents to the firms and give the average values of the equilibrium prices, hourly salary and wealth per agent. We have observed that the standard deviation over the 50 simulations is usually small, typically less that 5%.

**Fig 2:**
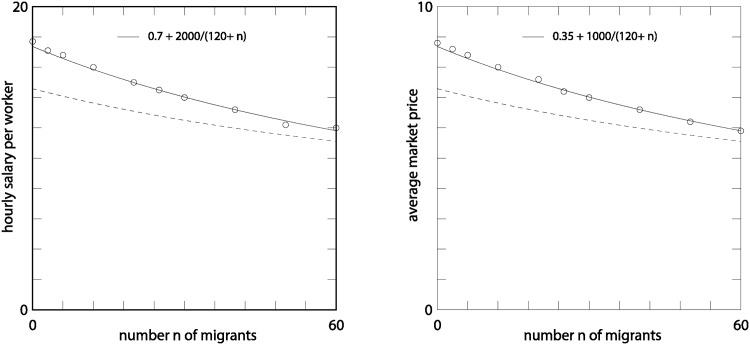
Overview of the values of the equilibrium hourly salary per worker and price of goods as a function of *n*, the number of migrants added to the reference economy with *N* = 120 and *M* = 30. The values for *n* = 0 correspond to the equilibrium values before adding the migrants. The dashed lines corresponds to the predictions of Eqs (31) and (33), respectively.

The average hourly salary per worker (there are *N* + *n* of them) and average price once the new equilibrium state is reached is shown in Fig 2. Here we consider sets of immigrants of sizes *n* = 3, *n* = 6, …*n* = 60. These sizes may appears to be small but, as discussed above, the results scale well to larger systems. For practical purpose, the quantity *n*/*N* is most relevant and amounts here to 2.5%, 5%, …, 50%. We observe a clear decrease of the new equilibrium prices and hourly salaries. This drop becomes larger as the size of the immigration wave increases. A fit for the equilibrium hourly salary *s* can be proposed, with the expression

$$\begin{array}{c} s\left(n\right)=an^{-1}+b \end{array}$$

with *a* ≈ 2000 and *b* ≈ 0.7. This power law behavior is actually compatible with the mean-field theory proposed in the next section, not exaclty those predicted by the theory (difference between the dashed and solid lines). Note also that Eq (33) shows that these coefficients *a* and *b* are not universal and depend on *N*, *M* and the investment strategy.

The same fit, but with coefficient divided by two does accurately reflect the price *p* as a function of *n*. The reason why *s*(*n*) and *p*(*n*) are multiples of each other, up to fluctuations, is explained in the next section, based on our mean-field, representative agent approach.

**Fig 3:**
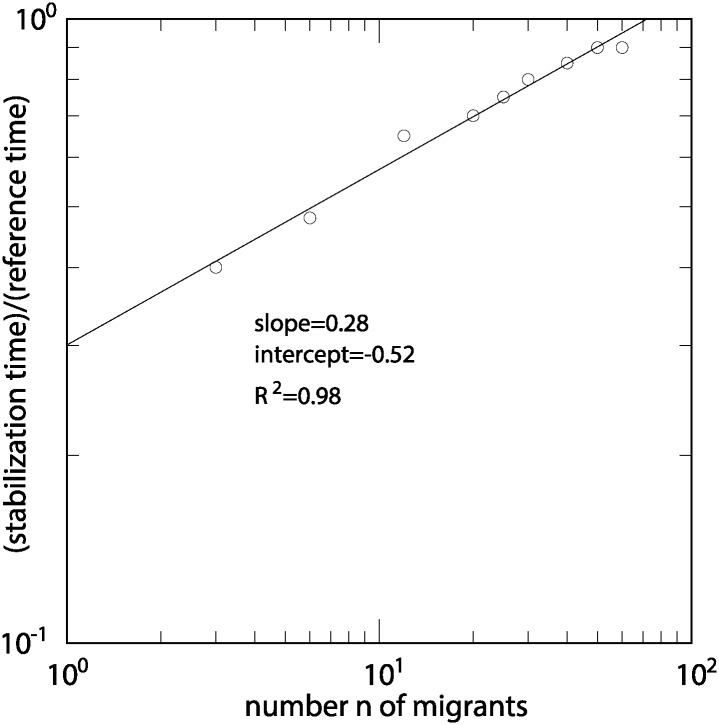
Number of iterations needed to reach a new equilibrium as a function of the number *n* of added immigrants.

The number of iterations *t*_*n*_ needed for the system to reach a new equilibrium point after the *n* immigrants have been added is another way to quantify the importance of the perturbation: during this stabilization period, the price and salary are subject to continuous changes that are the sign of a fragile and disturbed economy, which is negative for the whole society. We found that the time to stabilize the economy to its new equilibrium value increases with the size *n* of the perturbation. To give a better idea of the importance of the duration of this adjustment period, we compare it with the time the reference economy took to stabilize from its random initial condition. As discussed in section describing our reference economy, this time corresponds to about *T* = 20 model iterations. In Fig 3 we show the ratio *t*_*n*_/*T* in a log-log plot which suggests that *t*_*n*_ obeys the power law

$$\begin{array}{c} t_{n}=\alpha Tn^{\beta } \end{array}$$ (17)

with *β* = 0.28 and *α* = 10^−0.52^ = 0.3. These values show that *t*_*n*_ is a function that grows slower than linearly, suggesting that it is better to integrate all the migrants at once rather than step by step.

Finally, we have observed that the average wealth per agent is not much affected by the addition of immigrants to the economy [27].

## The representative agents approach

We now consider a description of the above multi-agent model with only two representative agents, one for the workers and one for the firm. This description assumes that all agents have the same cash and salary, and all the firms have the same man-power and the same number of customers. For this reason, we also refer to this approach as a mean-field approximation of the actual system. This simplification is made possible by the bipartite relation between the agents.

In this case, the equilibrium solution of the market (salary, price, wealth) can be obtained analytically as a function of the market size, *i.e.* the numbers of workers and firms. The behavior we obtain can then be compared to the multi-agent simulations to test the quality of the two-representative-agent approximation.

We denote *c*(*t*) the total wealth of all *N* workers in the systems at time *t*, and *C*(*t*) the total wealth of all *M* firms. We will therefore assume that our representative agents have a wealth *c*(*t*) and *C*(*t*), respectively.

As before, we define *λ* and *μ* as the fraction of respectively *c*(*t*) and *C*(*t*) that are invested for buying goods and paying salaries.

To go from iteration *t* to *t* + 1, the representative worker buys all the goods *G* produced at the previous iteration. Let us assume that the representative firm gives a salary *s*(*t*) to that worker. Then, this worker buys goods for an amount *a*(*t*). The dynamics can be expressed as

$$\begin{array}{ccc} c(t+1) & = & c(t)+s(t)-a(t)\\ C(t+1) & = & C(t)-s(t)+a(t). \end{array}$$ (18)

Eq (18) shows that *c*(*t*) + *C*(*t*) is constant over time, as it should, and that

$$\begin{array}{c} c(t)+C(t)=c(0)+C(0). \end{array}$$ (19)

In order to reach a steady state, *c*(*t* + 1) = *c*(*t*) and *C*(*t* + 1) = *C*(*t*), we need to have equality between the amount of salary and purchase.

$$\begin{array}{c} a(t)=s(t). \end{array}$$ (20)

From the model definition we have (the salary is received before the purchase)

$$\begin{array}{c} a(t)=\lambda (c(t)+s(t))\;s(t)=\mu C(t) \end{array}$$ (21)

where *λ* and *μ* are the investment fraction of the representative worker and firm. The condition *a*(*t*) = *s*(*t*) implies that

$$\begin{array}{c} s(t)(1-\lambda )=\lambda c(t)\;s(t)=\mu C(t) \end{array}$$ (22)

which requires that

$$\begin{array}{c} c\left(t\right)=\frac{\mu (1-\lambda )}{\lambda }C\left(t\right). \end{array}$$ (23)

Since, in addition, we have *C*(*t*) = *C*(0) + *c*(0) − *c*(*t*) we obtain that

$$\begin{array}{c} c\left(t\right)=\frac{\mu (1-\lambda )}{\lambda }\left(C\left(0\right)+c\left(0\right)-c\left(t\right)\right). \end{array}$$ (24)

Therefore

$$\begin{array}{c} c\left(t\right)\left(1+\frac{\mu (1-\lambda )}{\lambda }\right)=\frac{\mu (1-\lambda )}{\lambda }\left[C\left(0\right)+c\left(0\right)\right] \end{array}$$ (25)

and, by denoting the quantities at large time with the subscript ∞, we get

$$\begin{array}{c} c_{\infty }=\frac{\mu (1-\lambda )}{\lambda +\mu (1-\lambda )}\left[C\left(0\right)+c\left(0\right)\right]\;C_{\infty }=\frac{\lambda }{\lambda +\mu (1-\lambda )}\left[C\left(0\right)+c\left(0\right)\right] \end{array}$$ (26)

from which we also obtain

$$\begin{array}{c} \frac{c_{\infty }}{C_{\infty }}=\frac{\mu (1-\lambda )}{\lambda } \end{array}$$ (27)

and

$$\begin{array}{c} a_{\infty }=s_{\infty }=\mu C_{\infty }=\frac{\lambda \mu }{\lambda +\mu (1-\lambda )}\left[C\left(0\right)+c\left(0\right)\right] \end{array}$$ (28)

We can now consider how the above quantities depend on the size of the market, namely the numbers *N* of workers and *M* of firms.

We define the average cash per agent as

$$\begin{array}{c} c\left(0\right)=N\bar{c}\left(0\right)\;C\left(0\right)=M\bar{C}\left(0\right) \end{array}$$ (29)

$$\begin{array}{c} {\bar{c}}_{\infty }=\frac{\mu (1-\lambda )}{\lambda +\mu (1-\lambda )}\frac{M\bar{C}\left(0\right)+N\bar{c}\left(0\right)}{N}\;{\bar{C}}_{\infty }=\frac{\lambda }{\lambda +\mu (1-\lambda )}\frac{M\bar{C}\left(0\right)+N\bar{c}\left(0\right)}{M}. \end{array}$$ (30)

These relations suggest that the average wealth remains constant if the number of firms increases in proportion to the number of workers.

Furthermore, the number of goods produced at each iteration, *G*(*H*), is a function of the total number *H* = *Nh* of hours spent by the workers for the firms. The price *p* is defined as

$$\begin{array}{c} p_{\infty }=\frac{a_{\infty }}{G(H)}=\frac{\lambda \mu }{\lambda +\mu -\lambda \mu }\frac{M\bar{C}\left(0\right)+N\bar{c}\left(0\right)}{G(Nh)} \end{array}$$ (31)

From this relation we see that the price will drop if *N* increases and *G*(*Nh*) ∝ *N*, as we assumed in the numerical model. On the other hand, increasing *M* with *N* constant will lead to an increase of the price. With constant values of *N*, *M*, *λ* and *μ*, we obtained that *p*_∞_ = *an*^−1^ + *b*, as used to fit the data in Fig 2. But the value predicted by Eq (31) are *a* = 625 and *b* = 2.08, which differ from the values found to fit the data in Fig 2.

We also see that the individual salary

$$\begin{array}{c} s_{\infty }/N=\frac{\lambda \mu }{\lambda +\mu -\lambda \mu }\left(\frac{M}{N}\bar{C}\left(0\right)+\bar{c}\left(0\right)\right) \end{array}$$ (32)

decreases as *N* increases or increases as *M* increases.

The hourly salary is then *s*_∞_/(*hN*) where *h* the number of hours given by each worker. We then have the relation that

$$\begin{array}{c} \frac{s_{\infty }}{Nh}=p_{\infty }\frac{Nh}{G(Nh)}. \end{array}$$ (33)

Note also that the purchasing power *Π* of each individual can be defined as how much goods one can buy with one’s salary *s*_∞_/*N*, namely

$$\begin{array}{c} \Pi =\frac{s_{\infty }}{Np_{\infty }}=G\left(Nh\right)\frac{s_{\infty }}{Na_{\infty }}=\frac{G(Nh)}{N}. \end{array}$$ (34)

Therefore, for *G*(*Nh*) ∝ *Nh*, the purchasing power is constant for any size of the market. Remember that in the numerical model we arbitrarily defined that

$$\begin{array}{c} G(Nh)=2Nh \end{array}$$

assuming appropriate units to measure the amount of goods. From Eq (33), this choice yields *s*_∞_ = 2*p*_∞_, as also observed in the simulations (see Fig 2).

We can further compare the predictions of the above mean-field approach with the simulation of the multi-agent model, by setting *N* = 120 workers and *M* = 30 firms. The initial wealth of the workers and firms are *c*(0) = 12,000 and *C*(0) = 30,000, respectively, and the investment parameters are set to *λ* = *μ* = 0.5. These figures correspond to the reference economy described above.

As can be seen from Table 2, the wealths are consistent with the conservation law

$$\begin{array}{c} 120\times {\bar{c}}_{\infty }+30\times {\bar{C}}_{\infty }=120\times \bar{c}\left(0\right)+30\times \bar{C}\left(0\right). \end{array}$$

The proposed representative agent approach predicts the wealth of the firms, the price and hourly salary with an error below 20%. We observe that the firms are poorer within the mean-field approach than they are in the full model. Also the wealth of the workers is overestimated by a factor almost 2. These deviations probably reflect the local variations due to the structure of chosen random interaction network. They certainly call for further investigation.

**Table 2:** Comparison of the mean-field approximation and the actual model simulation. Here we have *N* = 120 workers, *M* = 30 firms, with investment parameters *λ* = *μ* = 1/2 and initial wealths *c*(0) = 12,000 and *C*(0) = 30,000. The subscript ∞ denotes the value at the equilibrium. The last column gives the average hourly salary, assuming *h* = 8 hours of work per day and per workers.

	${\bar{c}}_{\infty }$	${\bar{C}}_{\infty }$	*p*_∞_	*s*_∞_/(*h* × *N*)
initial	100	1000	-	-
Mean-field	116.7	933.33	7.29	14.58
Simulation	68.8	1124.8	8.8	17.7

Fig 2 shows, as a function of *n*, the behavior of Eqs (33) and (31), for *N* = 120 and *M* = 30. These equations predict a dependence as *b* + *a*/*n* which is well obeyed by the simulation data, but with different coefficients *a* and *b* than found with the mean-field approach.

Eq (30) suggests a 1/*n* dependence of the workers’ wealth as a function of *n* and a linear increase of the firms’ wealth. Neither of these two predictions are verified in the numerical simulations in which both workers’ and firms’ wealths seem not correlated to the number of added immigrants.

## Conclusion

In this paper we have proposed a theoretical framework to study how a stable economy reacts to a perturbation of increasing size. Our economical model is a multi-agent system on a dynamical complex network, and it implements a goods market coupled to a labor market. It has very few adjustable parameters which makes it quite robust. The properties of this virtual market is that prices and salaries are emerging properties of the dynamics, reflecting the global wealth of the market and its capability to produce goods, as well as the topology of the transactions.

We have applied this model to study the impact of immigration on a stable economy. To our knowledge, this is the first attempt of that type in this direction. Of course our approach is over-simplified with respect to existing socio-economical and political situations, and it is based on hypotheses that may not be guaranteed: for example each immigrant immediately finds a job, and comes with some personal assets. Despite this idealized situation, the integration perturbs the systems in a macroscopic way. Probably, the most negative part is the fact that the market needs to readjust to the new situation, and the resulting instability may be a factor of risk to the economy. But besides that, the quality of life at the new equilibrium point is identical to the previous one: the salaries have decreased, but so did the prices.

An interesting part of our model is the functional relation it predicts between the numbers of immigrants and the variation of price, wage and wealth. We have observed power law dependencies that, if found correct in real situation, would be a very relevant indication to manage an immigration event.

Our model, due to its structure, can easily be extended in many directions, provided that more socio-economical knowledge is available. Our results obviously need to be confronted to some real data. We hope that this modest contribution will stimulate further research in this direction.

One could for instance find some elements of validation in the large immigration events that occurred in recent history and that showed that, after a transient period, immigration has a positive return on the economy. For instance in 1980, Fidel Castro let 120,000 people move to Florida, increasing the active population of Miami by 7%. Five years later, the economical situation of Miami was better than many other comparable American cities [28].

In agreement with several existing economical studies, our model also indicates that, in the long run, immigration is rather neutral with respect to the purchase power of consumers. A key point is obviously to ensure that the newcomers quickly find a job and become an integral part of the economic system. Government investments to facilitate such integration are recognized as a central issue, in particular by the Organization for Economic Co-operation and Development (OECD) [29].
